# NNAN: Nearest Neighbor Attention Network to Predict Drug–Microbe Associations

**DOI:** 10.3389/fmicb.2022.846915

**Published:** 2022-04-11

**Authors:** Bei Zhu, Yi Xu, Pengcheng Zhao, Siu-Ming Yiu, Hui Yu, Jian-Yu Shi

**Affiliations:** ^1^School of Life Sciences, Northwestern Polytechnical University, Xi’an, China; ^2^Department of Computer Science, The University of Hong Kong, Hong Kong, China; ^3^School of Computer Science, Northwestern Polytechnical University, Xi’an, China

**Keywords:** deep learning, bipartite graph network, link prediction, drug–microbe association, attention matrix

## Abstract

Many drugs can be metabolized by human microbes; the drug metabolites would significantly alter pharmacological effects and result in low therapeutic efficacy for patients. Hence, it is crucial to identify potential drug–microbe associations (DMAs) before the drug administrations. Nevertheless, traditional DMA determination cannot be applied in a wide range due to the tremendous number of microbe species, high costs, and the fact that it is time-consuming. Thus, predicting possible DMAs in computer technology is an essential topic. Inspired by other issues addressed by deep learning, we designed a deep learning-based model named Nearest Neighbor Attention Network (NNAN). The proposed model consists of four components, namely, a similarity network constructor, a nearest-neighbor aggregator, a feature attention block, and a predictor. In brief, the similarity block contains a microbe similarity network and a drug similarity network. The nearest-neighbor aggregator generates the embedding representations of drug–microbe pairs by integrating drug neighbors and microbe neighbors of each drug–microbe pair in the network. The feature attention block evaluates the importance of each dimension of drug–microbe pair embedding by a set of ordinary multi-layer neural networks. The predictor is an ordinary fully-connected deep neural network that functions as a binary classifier to distinguish potential DMAs among unlabeled drug–microbe pairs. Several experiments on two benchmark databases are performed to evaluate the performance of NNAN. First, the comparison with state-of-the-art baseline approaches demonstrates the superiority of NNAN under cross-validation in terms of predicting performance. Moreover, the interpretability inspection reveals that a drug tends to associate with a microbe if it finds its top-*l* most similar neighbors that associate with the microbe.

## Introduction

The human microbiome refers to all the microbes associated with a human body, including bacteriophages, archaea, bacteria, eukaryotes, and fungi (Lynch and Pedersen, 2016). To assess the diversity and functions of the human microbiome, the Human Microbiome Project (HMP) was supported by the National Institutes of Health (NIH) from 2007 to 2016 (Turnbaugh et al., 2007). HMP provided a complete description of the microbiome in five tissues of the human body, including skin, gut, nostrils, vagina, and mouth (Aagaard et al., 2013). Human microbes have been verified for their close associations with human health by cell experiments, animal experiments, epidemiological studies, clinical case studies (Schwabe and Jobin, 2013; Lynch and Pedersen, 2016), etc. Previous works have revealed that abnormal microbe communities lead to metabolic disorders [e.g., non-alcoholic fatty liver disease (Younossi et al., 2016), obesity, and diabetes mellitus (Jaacks et al., 2019; Zheng et al., 2018)]. Oral drug administration is a typical treatment. Many drugs, however, can be metabolized by human microbes, and the drug metabolites would significantly alter pharmacological effects and result in low therapeutic efficacy for patients. For example, after being modified by gut microbes, the compounds can lead to their activation [e.g., *salicylazosulfapyridine* (Sousa et al., 2014)] or inactivation [e.g., inactivation of the cardiac drug digoxin by the intestinal actinomycete *Eggerthella lenta* (Haiser et al., 2013)], or induce toxicity [e.g., 70% toxicity of Brivudine may be attributed to intestinal microorganisms (Zimmermann et al., 2019b)]. The persistent findings of microbiome-induced individual pathogenesis, phenotypes, and treatment responses boost the microbiome to be an integral part of precision medicine (Kashyap et al., 2017). Therefore, drug–microbe association (DMA) prediction is of great significance for therapy and medicine development. However, the acquisition of DMAs needs a large scale of assays with high costs, low efficiency, and culturing limitations, and that are time-consuming. To identify DMAs rapidly and effectively, machine learning methods, especially deep learning-based methods, have attracted many scientists due to their inspiring applications in other areas [e.g., predicting microbe–disease associations (He et al., 2018; Peng et al., 2018), drug–drug interactions (Yu et al., 2021a), lncRNA–miRNA interactions (Zhang L. et al., 2021), and lncRNA–protein interactions (Lihong et al., 2021; Zhou et al., 2021)].

In recent years, researchers have applied Graph Attention Network [GAT (Velickovic et al., 2018)] to bioinformatics with remarkable results. For instance, Zhang Z. et al. (2021) used fragments containing functional groups to represent molecular maps for molecular property prediction through a fragment-oriented multi-scale graph attention model. Bang et al. (2021) made the prediction of polypharmacy side effects with enhanced interpretability based on graph feature attention network. Constructing a bipartite network is the most popular approach to represent associations between two types of nodes. The prediction problem of DMA can then be transformed into a link prediction problem in a bipartite graph network. However, few models predict DMAs through bipartite graph networks. For example, EGATMDA (Long et al., 2020b) used the drug–disease–microbe perspective to predict the DMAs, which does not show a direct relationship between drugs and microbes and may contain noise. HMDAKATZ (Zhu et al., 2019) predicted the interactions between drugs and microbes based on the Katz (1953); the disadvantage of this method in the node’s information transmission (i.e., a node with a high central value transmits its high influence to all its neighbors) may not be appropriate in real life. GCNMDA (Long et al., 2020a) used GCN, random walk with restart, and GAT to learn node features, which relies on the parameter “step size” when using the restart random walk algorithm. HNERMDA (Long and Luo, 2020) learned the drug–microbe heterogeneous network information by metapath2vec measure, which considered the type of nodes in the meta-path-based random walk but the skip-gram does not treat them differently during training.

In the field of drug–target interaction prediction, there is a widely accepted assumption that structurally similar drugs tend to interact with the same target (Khalili et al., 2012). Analogously, we anticipate that if a drug (d_*x*_) can associate with a microbe (b_*p*_), the other drugs associated with the same microbe (b_*p*_) are usually the first *l* nearest neighbors of the drug (d_*x*_). Therefore, we propose a new model, Nearest Neighbor Attention Network (NNAN), which aggregates the information from nodes’ neighbors according to their entity types and maps them into a unified embedding space for further predicting potential DMAs. The comparison with state-of-the-art methods on two different databases demonstrates the superiority of our NNAN. Moreover, its interpretability is illustrated and validates our assumption. Finally, the case study assesses its ability to find potential associations between drugs and microbes. In general, our contribution is as follows:

• We make use of three networks: drug–drug similarity network, microbe–microbe similarity network, and a drug–microbe bipartite graph network. Imitate the idea of KNN [K-Nearest-Neighbor (Cover and Hart, 1967)] to learn the substructures of the bipartite graph network, which can promote the accuracy of link prediction.
• We follow the idea of GAT and use multiple DNNs to learn the weights of embedding features to improve the screening efficiency of potential associations.
• In a quantitative way, we verify the hypothesis that “If a drug can associate with a microbe, the other drugs that associate with the microbe are usually the first *l* nearest neighbors to the drug.”

## Materials and Methods

**FIGURE 1:**
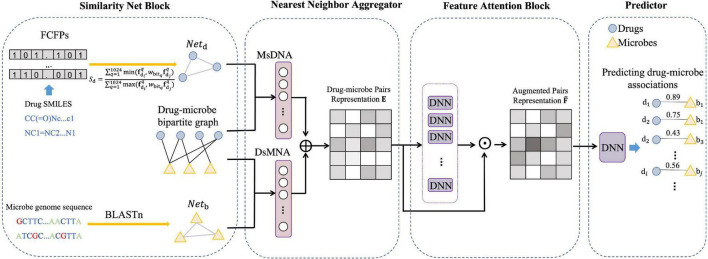
The overall framework of NNAN for drug–microbe association prediction.

In this section, we describe a model for predicting DMAs in a bipartite graph network, named NNAN as shown in Figure 1. It consists of four components: a similarity network constructor, a nearest-neighbor aggregator, a feature attention block, and a predictor. Firstly, the similarity network constructor is mainly used to build a drug similarity network and a microbe similarity network (section “Similarity Networks” for details). Secondly, the nearest-neighbor aggregator generates the embedding representations of drug–microbe pairs by integrating drug neighbors and microbe neighbors of each drug–microbe pair in the network (section “Nearest-Neighbor Aggregator for Drug–Microbe Pair Embeddings” for details). Thirdly, the feature attention block evaluates the importance of each dimension of drug–microbe pair embedding by a set of ordinary multi-layer neural networks (section “Feature Attention Block” for details). Finally, we make use of a fully-connected deep neural network as a binary classifier to predict potential DMAs.

### Similarity Networks

#### Drug Similarity Network

We calculate drug similarities by the following steps. First, drugs are represented by Functional-Class Fingerprints [FCFPs (Rogers and Hahn, 2010)], which is the generalized version of Extended-Connectivity Fingerprints [ECFPs (Rogers and Hahn, 2010)] with more attention to atom functions. The FCFPs is implemented by RDKit (Landrum, 2010). Second, the similarity between drug d_*i*_ and drug d_*j*_ is calculated by the Tanimoto coefficient (Rogers and Tanimoto, 1960) as follows:


(1) $$S\,\left({\text{d}}_{i},{\text{d}}_{j}\right)=\frac{{\text{f}}_{{\text{d}}_{i}}\cdot {\text{f}}_{{\text{d}}_{j}}}{||{\text{f}}_{{\text{d}}_{i}}||+||{\text{f}}_{{\text{d}}_{j}}||-{\text{f}}_{{\text{d}}_{i}}\cdot {\text{f}}_{{\text{d}}_{j}}}$$

where **f**_d_*i*__ and **f**_d_*j*__ represent the FCFPs vector of drug d_*i*_ and drug d_*j*_, respectively, ||⋅|| indicates the norm of the vector.

Fingerprint similarity provides intuitive results: why the two molecules have been determined to be similar, but this transparency tends to vanish completely when molecular fingerprints are used as input to machine learning models. Inspired by the similarity maps (Riniker and Landrum, 2013), we calculate the contribution of each atom to the similarity between two molecules. To make it easier to distinguish the drugs, we regard d_*i*_ as a reference drug, d_*j*_ as a comparison drug, and *S*(d_*i*_,d_*j*_) as the base similarity of this drug pair. The RDKit will automatically number each atom of the comparison drug d_*j*_ (*K* = {0,1,…,*t*−1}). Then, we remove the atoms of the comparison drug one by one in the order of the atomic numbers to form multiple new comparing drugs (${\text{d}}_{j}^{k},k\in K,K=\left\{0,1,\ldots ,t-1\right\}$). We calculate the new similarity between the reference drug (d_*i*_) and the new comparison drug (${\text{d}}_{j}^{k}$), and regard the difference between the new similarity and the base similarity as the weight ($w{}_{j}^{k}$) of each removed atom. The weight $w{}_{j}^{k}$ is formulated as:


(2) w=jk|S(di,dj)-S(di,djk)|

We set the dimension of the FCFPs vector to 1,024 bits, of which the non-zero bits indicate the occurrences of drug feature substructures. To obtain the weight of each non-zero bit, we add up the weights of all the atoms contained in the feature substructure:


(3) $$w_{{\text{bit}}_{q}}=S\,U\,M_{q}\,\left(w_{j}^{k}\right)$$

where *w*_*bit*_*q*__ denotes the weight of the *q*_*th*_ dimensional bit of the FCFPs vector, and the function *SUM*_*q*_(⋅) denotes the sum of all the atomic weights contained in the feature substructure represented by the *q*_*th*_ dimensional bit of the FCFPs.

Then, the weighted Tanimoto similarity (Ioffe, 2010) between the reference drug and the comparison drug can be calculated as follows:


(4) $$S_{\text{d}}\,\left({\text{d}}_{i},{\text{d}}_{j}\right)=\frac{\sum_{q=1}^{1024}\text{min}\,\left({\text{f}}_{{\text{d}}_{i}}^{q},w_{{\text{bit}}_{q}}\,{\text{f}}_{{\text{d}}_{j}}^{q}\right)}{\sum_{q=1}^{1024}\text{max}\,\left({\text{f}}_{{\text{d}}_{i}}^{q},w_{{\text{bit}}_{q}}\,{\text{f}}_{{\text{d}}_{j}}^{q}\right)}$$

where ${\text{f}}_{{\text{d}}_{i}}^{q}$ and ${\text{f}}_{{\text{d}}_{j}}^{q}$ denote the *q*_*th*_ dimension of the FCFPs vectors for the reference drug and the comparison drug.

Based on drug similarities, we can build a drug similarity network *Net*_d_, where nodes are drugs. There are edges between the drugs if these drugs associate with the same microbe; the edges are weighted by drug similarities.

#### Microbe Similarity Network

To calculate microbe similarities, we use BLAST (Altschul et al., 1990) to make pairwise alignments of microbial genomes. Specifically, the main function of BLAST is to discover local similarity regions between sequences and then use the local sequence alignment algorithm (Smith and Waterman, 1981) to calculate the similarity. For example, ${\text{G}}_{\text{A}}={\text{g}}_{\text{A}}^{1}\,{\text{g}}_{\text{A}}^{2}\,\ldots \,{\text{g}}_{\text{A}}^{n}\,\mathrm{and}\,{\text{G}}_{\text{B}}={\text{g}}_{\text{B}}^{1}\,{\text{g}}_{\text{B}}^{2}\,\ldots \,{\text{g}}_{\text{B}}^{m}$ are the genome sequences of microbe A and microbe B, where *n* and *m* are the lengths of sequences G_A_ and G_B_, respectively. BLAST creates the scoring matrix **H**_(**n**+**1**)×(**m**+**1**)_ and makes the first row and column elements zero. The formula for the element **H**_*ij*_(**H**_*ij*_ ∈ **H**_(**n**+**1**)×(**m**+**1**)_,*i* = 1,2,…,*n*;*j* = 1,2,…,*m*) in this scoring matrix is:


(5) Hi⁢j=max{Hi-1,j-1+ScoreHi-k,j-2Hi,j-k-20(g=Aig,BjScore=1;g≠Aig,BjScore=-1)

the highest value in the matrix **H**_(*n* + 1)×(*m* + 1)_ is chosen as *sw*(G_A_,G_B_). The similarity between microbes A and B is adopted by the same definition as Yamanishi et al. (2008), as follows:


(6) $$S_{\text{b}}\,\left(A,B\right)=\frac{s\,w\,\left({\text{G}}_{\text{A}},{\text{G}}_{\text{B}}\right)}{\sqrt{s\,w\,\left({\text{G}}_{\text{A}},{\text{G}}_{\text{A}}\right)\times s\,w\,\left({\text{G}}_{\text{B}},{\text{G}}_{\text{B}}\right)}}$$

Based on microbe similarities, we can build a microbe similarity network *Net*_b_, where nodes are microbes. There are edges between the microbes if these microbes associate with the same drug; the edges are weighted by microbe similarities.

### Nearest-Neighbor Aggregator for Drug–Microbe Pair Embeddings

In this section, inspired by the idea of KNN [K-Nearest-Neighbor (Cover and Hart, 1967)], we learn the substructures of the bipartite graph network to obtain the embedding representations of drug–microbe pairs.

**FIGURE 2:**
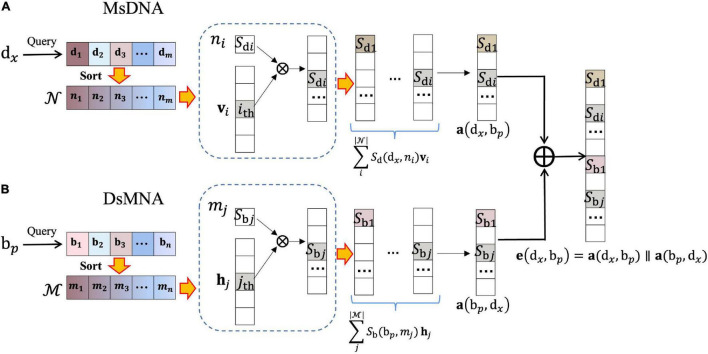
Nearest-Neighbor Aggregator block. **(A)** Microbe-specific drug neighbor aggregator (MsDNA); the embedding representation of the unidirectional edge, which is from the drug d_*x*_ to the microbe b_*p*_. **(B)** Drug-specific microbe neighbor aggregator (DsMNA); the embedding representation of the unidirectional edge, which is from the microbe b_*p*_ to the drug d_*x*_, where 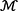
*^x^*⊆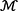 is a set of instantiated keywords, 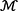
*^x^* denotes the neighbors of microbe b_*p*_ in the *Net*_b_. *S*_b_(b_*p*_,*m*_*j*_) denotes the similarity of b_*p*_ and *m_j_*. **h**_*j*_ is the corresponding one-hot encoding vector of *m_j_*.

First, we construct the drug–microbe bipartite graph network, G=(D,B,E), where D={d_1_,d_2_,…,d_*m*_} represents *m* drugs, B={b_1_,b_2_,…,b_*n*_} represents *n* microbes, and each edge (e_*ij*_) in edge set E connects two nodes that belong to two different sets of vertexes (i.e., *i* in D, *j* in B). We regard the DMAs as bidirectional links. That is, e_d_*x*_→b_*p*__ denotes the edge pointing from the drug d_*x*_ to the microbe b_*p*_, and *e*_*b_p_→d_x_*_ denotes the edge pointing from the microbe b_*p*_ to the drug d_*x*_. Correspondingly, the nearest-neighbor aggregator contains two blocks (Figure 2), the microbe-specific drug neighbor aggregator (MsDNA), and the drug-specific microbe neighbor aggregator (DsMNA). Due to their architectures being similar, we only illustrate the MsDNA block in this section.

Microbe-specific drug neighbor aggregator (Figure 2A) contains a virtual key dictionary; 𝒩={*n*_1_,*n*_2_,…,*n*_*m*_} indicates all the drugs. In the dictionary, we imitate the idea of KNN to learn the substructures of the bipartite graph network, where virtual keys are sorted by their semantic nearest neighbors. In simple terms, *n_1_* denotes d_*x*_ itself, its nearest neighbor is the second key, and the farthest neighbor is the last key. The embedding representation of the edge, which is from drug d_*x*_ to microbe b_*p*_, is formulated as follows:


(7) $$\text{a}\left({\text{d}}_{x},{\text{b}}_{p}\right)=\sum_{i}^{\left|𝒩\right|}S_{\text{d}}\left({\text{d}}_{x},n_{i}\right){\text{v}}_{i}\left(ifn_{i}\notin {𝒩}^{p},S_{\text{d}}\left({\text{d}}_{x},n_{i}\right)=0\right)$$

where 𝒩^*p*^⊆𝒩 is a set of instantiated keywords, and 𝒩^*p*^ denotes the neighbors of d_*x*_ in the *Net*_d_. *S*_d_(d_*x*_,*n*_*i*_) denotes the similarity of d_*x*_ and *n_i_*, and **v**_*i*_ is the corresponding one-hot encoding vector of *n_i_* (i.e., the one-hot encoding has a non-zero value only in the *i*_*th*_ element, and all other position elements are zero).

Similarly, DsMNA (Figure 2B) makes the single directional embedding representation from b_*p*_tod_*x*_ as **a**(b_*p*_,d_*x*_). Then, the representation of drug–microbe pair could be encoded as


(8) $$\text{e}\,\left({\text{d}}_{x},{\text{b}}_{p}\right)=\left[\text{a}\,\left({\text{d}}_{x},{\text{b}}_{p}\right)∥\text{a}\,\left({\text{b}}_{p},{\text{d}}_{x}\right)\right]$$

where **e**(d_*x*_,b_*p*_) is generated *via* the concatenation of bidirectional embedding, and ∥ is the concatenation operation. All the embedding representations of drug–microbe pairs could stack as a matrix **E**_*k*×*g*_, where *k* is the number of all the drug–microbe pairs and *g* is the dimension of each embedding. The nearest-neighbor aggregator effectively learns the bipartite graph substructures, and **E**_*k*×*g*_ will be input into a feature attention block to select crucial features for achieving a better DMA prediction.

### Feature Attention Block

**FIGURE 3:**
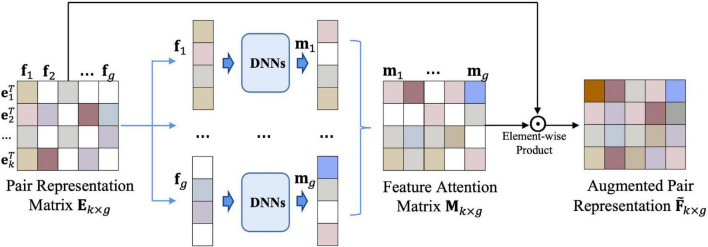
Feature attention block. Input the representation matrix **E**_*k*×*g*_ into a set of DNNs, then we obtain an attention matrix **M**_*k*×*g*_of drug–microbe embedding features. After the element-wise product operation of **M**_*k*×*g*_ and **E**_*k*×*g*_, the final feature matrix ${\tilde{\text{F}}}_{k\times g}$ of the drug–microbe pairs is obtained.

To improve the performance of the prediction, we build the feature attention block (Figure 3) for updating the embedding of drug–microbe pairs.

Recall the equation of output feature representation in GAT (Velickovic et al., 2018):


(9) $$\vec{h_{i}^{\prime }}=\sigma \,\left(\sum_{j\in {𝒦}_{i}}\alpha_{i\,j}\,\text{W}\,\vec{h_{j}}\right)$$

where σ is a nonlinear activation function, 𝒦^*i*^ is the first-order neighbors of node *i* (including *i*), α_*ij*_ is the coefficients computed by the attention mechanism, and **W** is a weight matrix. To make equation (9) easier to understand. We compute the coefficients as:


(10) $$\sum_{j\in {𝒦}_{i}}\alpha_{i\,j}=\tilde{\text{A}}⊙\text{M}$$

where $\tilde{\text{A}}=\text{A}+\text{I}$ is the adjacency matrix of the undirected graph G with added self-connections (Kipf and Welling, 2017), ⊙ is the element-wise product operation, and **M** is the attention matrix. Then, the layer-wise propagation rules in GAT can be formulated as:


(11) $${\text{H}}^{\left(l+1\right)}=\sigma \,\left(\left(\tilde{\text{A}}⊙\text{M}\right)\,{\text{H}}^{\left(l\right)}\,{\text{W}}^{\left(l\right)}\right)$$

where σ is a nonlinear activation function, and **W**^(*l*)^ is the weight matrix of the *l*_*th*_ neural network layer.

Inspired by the conception of the layer-wise propagation rules in GAT, we calculate the augmented representation matrix ${\tilde{\text{F}}}_{k\times g}$ by


(12) $${\tilde{\text{F}}}_{k\times g}={\text{E}}_{k\times g}⊙{\text{M}}_{k\times g}$$

where **E**_*k*×*g*_ is the representation matrix of the drug–microbe pairs obtained from the nearest-neighbor aggregator, **M**_*k*×*g*_ is an attention matrix of **E**_*k*×*g*_, and ⊙ is the element-wise product operation. We take the representation matrix **E**_*k*×*g*_ as a feature matrix **F** (**F**={**f**_1_,**f**_2_,…,**f**_*g*_}), which is composed of *g* column vectors (**f**_*i*_(*i* = 1,2,…,*g*)). The feature attention block mainly uses **M**_*k*×*g*_ to indicate the importance of features in the **E**_*k*×*g*_. Each feature dimension **f**_*i*_ can be labeled as “selected” or “discarded” in a hard way, or be associated with a probability to be selected in a soft way; we employ DNNs to model the mapping by


(13) $${\text{m}}_{i}=\text{DNNs}\,\left[{\text{f}}_{i}\right]$$

the DNN contains an input layer for each element of the feature dimension **f**_*i*_ and an output layer with sigmoid as its activation function.

In total, we build *k*×*g* DNNs to obtain **M**_*k*×*g*_. The final feature matrix ${\tilde{\text{F}}}_{k\times g}$ of the drug–microbe pairs is obtained after the element-wise product operation of **M**_*k*×*g*_ and **E**_*k*×*g*_. ${\tilde{\text{F}}}_{k\times g}$ is further fed into a predictor to achieve better predictive performance.

### Predictor

To implement the link prediction in the drug–microbe bipartite graph network, an ordinary DNN is utilized as the binary predictor that contains an input layer for the embedding representation of drug–microbe pairs, a hidden layer with ReLU as its activation function, and the two-neuron output layer with Sigmoid as its activation function. The output layer generates a probability that indicates the association likelihood of the drug and the microbe. The probability is formulated as:


(14) $$P=\varphi \,\left(ℱ\,\left(\text{ReLU}\,\left[ℱ\,\left(\tilde{\text{F}}\right)\right]\right)\right)$$

where φ is the sigmoid activation function, and ℱ(⋅) is the fully-connected layer.

The entire network of NNAN with the nearest-neighbor aggregator, feature attention weights, and DNN weights can be jointly optimized through the binary cross-entropy loss as follows:


(15) $$l\,o\,s\,s=\text{Y}\,\text{log}\,\left(𝒟\,\left(\tilde{\text{F}}\right)\right)+\left(1-\text{Y}\right)\,\text{log}\,\left(1-𝒟\,\left(\tilde{\text{F}}\right)\right)+\lambda \,ℛ\,\left(\theta \right)$$

where **Y** is the truth labels of drug–microbe pairs, 𝒟(⋅) is the DNN, θ denotes the weight parameters in the entire network, ℛ(⋅) is an L_2_-norm, and λ is coefficient of the regularization item.

## Experiments and Results

### Data

In our experiments, two databases are collected from MDAD (Sun et al., 2018) and Zimmermann et al. (2019a), respectively. The former work MDAD (Sun et al., 2018) investigated 5,505 clinically or experimentally DMAs between 1,388 drugs and 180 microbes. After removing redundant information, these association entries are grouped into Database 1, which contains 999 drugs, 133 microbes, and 1,708 DMAs.

The latter work (Zimmermann et al., 2019a) originally studied how 76 kinds of human gut bacteria metabolize 271 oral drugs, and found that 176 out of 217 drugs are significantly consumed by at least one bacteria strain. These associations are grouped into Database 2, which includes 176 drugs, 76 bacteria, and 4,194 associations (These two databases are shown in Table 1).

**TABLE 1:** The statistics of two databases.

	Drugs	Microbes	Associations
Database 1	999	133	1,708
Database 2	176	76	4,194

### Comparison

Since there are few existing approaches for predicting DMAs, we compare NNAN with three state-of-the-art methods, which were raised for bipartite link prediction.

• LAGCN (Yu et al., 2021b): A layer attention graph convolutional network for the drug–disease association prediction.

• NIMCGCN (Li et al., 2020): A neural inductive matrix completion with graph convolutional networks for miRNA–disease association prediction.

• GCNMDA (Long et al., 2020a): Predicting human microbe–drug associations *via* graph convolutional network with conditional random field.

To evaluate the performance of these methods, we regard the known DMA pairs as positive samples and unlabeled DMA pairs as negative samples (Peng et al., 2020; Li et al., 2022). We set up a 5-fold cross-validation scenario in which we randomly divide positive samples and negative samples into five groups, respectively. One group of positive samples and one group of negative samples are treated as test samples in turn for each round. The remaining groups are used for training purposes. Our model is trained by Gradient Descent Optimizer (Cauchy, 2009), with batch size 3,000 for 2,000 epochs, the initial learning rate is set to 0.9, and the regularization rate is set to 2e-4. We use AUROC (area under the receiver operating characteristic curve) and AUPRC (area under the precision-recall curve) as metrics to measure the DMA prediction performance. Moreover, we investigate the running time in terms of per epoch.

The comparison (Table 2) shows that NNAN obtains the best AUROC value (0.911) and the best AUPRC value (0.502) in Database 1. NNAN attains the next-highest AUROC value (0.902) and the best AUPRC value (0.840) in Database 2. To further present the performance of NNAN, we calculate the running time for one epoch of the baselines and NNAN, respectively. As presented, with the same computing equipment, NNAN takes the third-shortest running time in Database 1 and the shortest running time in Database 2. In general, we can see that NNAN are comparable in terms of AUROC, AUPRC, and computation time. It demonstrates that NNAN is superior to other methods on the databases we collected.

**TABLE 2:** The performance comparison of DMA prediction.

Method	Database 1			Database 2		
	AUROC	AUPRC	Time (s/epoch)	AUROC	AUPRC	Time (s/epoch)
LAGCN	0.861	0.323	**0.201**	**0.944**	0.721	0.021
NIMCGCN	0.778	0.156	19.076	0.815	0.720	0.721
GCNMDA	0.894	0.042	0.341	0.821	0.177	0.127
NNAN	**0.911**	**0.502**	0.649	0.902	**0.840**	**0.019**

*The highest value is indicated in bold, and the next highest value is underlined.*

### Interpretability of Nearest Neighbor Attention Network

How does the NNAN interpret the hypothesis that “If a drug can associate with a microbe, the other drugs that associate with the microbe are usually the first *l* nearest neighbors to the drug.”

The model has two significant advantages to enhance interpretability. First, each column vector **m_i_** of **M**_*k*×*g*_ indicates the global importance of each feature dimension **f**_*i*_. Moreover, the element-wise product between **E**_*k*×*g*_ and **M**_*k*×*g*_ generates the importance map of embedding features.

We first use the MsDNA in the nearest-neighbor aggregator block to show how the representation of drug–microbe pairs can provide intuitive hints, on which embedding features lead to the association. For the queried drug d_*x*_ to the microbe b_*p*_ of associated, non-zero cells in the embedding representation of **a**(*d*_*x*_,b_*p*_) stand for its attention values derived from the drugs commonly linking b_*p*_. Since the keys are sorted in descending order from the drug itself (*n_1_*) to the farthest neighbor (*n_m_*), the positions of non-zero cells are crucial to the final association.

**FIGURE 4:**
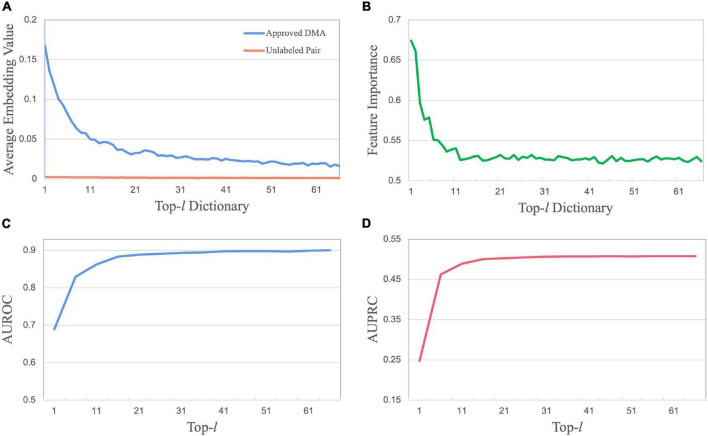
Mensurable clues of embedding features to the association outcome. **(A)** The distribution of embedding features along with the sorted drug neighbor keys. **(B)** The distribution of feature importance along with sorted node neighbor keys. **(C)** The predictive performance with top-*l* features concerning *l* in terms of AUROC. **(D)** The predictive performance in terms of AUPRC.

Take Database 1 as an example. By calculating two average embedding vectors for approved DMAs and unlabeled drug–microbe pairs, we obtained a distribution along with the drug key dictionary from *n_1_* to *n*_*66*_(Figure 4A). As illustrated, the significantly high values of embedding features occurring among the first *l* nearest neighbors reveal that a drug (d_*x*_) associated with a specific microbe (b_*p*_) can always find its top-*l* nearest neighbors among other drugs that associate with the same microbe. This observation demonstrates that a drug is possibly associated with the microbe if it has more non-zero value cells on the positions of the first *l* feature dimensions. This phenomenon could be caused by the fact that over 80% of approved drugs are of “follow-on” or “me-too” drugs. Due to high cost and high risk, the design of novel drugs, except for pioneer drugs, always starts from the structures of one or several existing drugs and then slightly modify them until meeting pharmacological needs (DiMasi and Faden, 2011). Analogously, the results of the DsMNA block along the microbe neighbor aggregator keys reveal that a microbe associated with a specific drug usually finds its near neighbors associated with the same drug.

Moreover, we illustrate how the feature attention matrix **M**_*k*×*g*_ can provide data-driven hints on which embedding features lead to the association. Since a high-value cell in **M**_*k*×*g*_ stands for a crucial feature dimension contributing to determine the association between a queried drug and a microbe, the importance **m**(*i*,:) of each feature **f**_*i*_ can be measured by the average of value entries in the *i*_*th*_ column of **M**_*k*×*g*_ (Figure 4B). The importance distribution along with the sorted drug neighbor keys illustrates that highly important features are usually located among the first *l* nearest neighbors. In addition, the predictive performance with top-*l* features concerning *l* is investigated (Figures 4C,D). The number of top features is tuned in the list {1, 6, 11, 16,…, 66}. As *l* is increasing to 16, the performance increases sharply in the top-*l* features. When *l* keeps increasing, the performance increases slowly, then even decreases at the greater value of *l*. Again, this illustration demonstrates that the selection of crucial features is significantly better than the set of all features.

In summary, both embedding feature matrix **E**_*k*×*g*_, which is generated by the nearest-neighbor aggregator, and its feature attention matrix **M**_*k*×*g*_ provide mensurable clues to the association outcome.

To complement the verification of the interpretability of NNAN, we selected one microbe (i.e., *Staphylococcus aureus*, which is a common causative agent of food poisoning) and one drug (i.e., *Hexyl gallate*, which has strong antimalarial activity against *Plasmodium falciparum*) from Database 1, and there was an association between them (de Lima Pimenta et al., 2013). We calculated the similarities between drugs using *Hexyl gallate* as the reference molecule and sorted the drugs in order of their similarity to *Hexyl gallate.* Then, we picked the top 10 drugs and checked whether these drugs were associated with *S. aureus* in Database 1. Finally, we found out that 8 out of the top 10 ranked drugs for *Hexyl gallate* are associated with *S. aureus* (Table 3).

**TABLE 3:** The associations among *Staphylococcus aureus* and ten drugs.

Drug name	Rank	Association	Drug name	Rank	Association
*Octyl gallate*	1	Yes	*Tannic acid*	6	No
*Butyl gallate*	2	Yes	*Tea tree oil*	7	Yes
*Octadecyl gallate*	3	Yes	*Pentagalloylglucose*	8	Yes
*Ethyl gallate*	4	Yes	*4-Ethylcatechol*	9	No
*Methyl gallate*	5	Yes	*Hamamelitannin*	10	Yes

*These ten drugs are ranked in order of their similarity to Hexyl gallate.*

From Table 3, it is clear that a drug tends to associate with a microbe if it finds its top-*l* near neighbors associate with the same microbe. Moreover, the higher the ranks of its top-*l* near neighbors are, the more possible it is to associate with the microbe. This conclusion would be helpful to screen drug-like molecules.

## Case Study of Novel Prediction

To further confirm the effectiveness of NNAN, we apply our model on one microbe (i.e., *Bacteroides fragilis*) in Database 2 as a case study. Bacteroides are the major human colonic commensal microbes (Kuwahara et al., 2004). Although *B. fragilis* is rare in comparison to other Bacteroides species, it is the most prevalent clinical isolation of the genus (Salyers, 1984). Thus, we select *B. fragilis* for the case study experiment.

Nearest neighbor attention network predicts potential associations between drugs and *B. fragilis* by scoring drug–microbe pairs (probability). The higher the score, the more likely the association between the drugs and *B. fragilis* exists. In the case study, we verified whether NNAN could find out potential linkages between *B. fragilis* and drugs. According to the ranking of potential DMAs, we validated the top 10, 20, and 50 predicted candidate drugs by a literature search. Eventually, the validation indicates that 10, 17, and 38 out of the top 10, 20, and 50 predicted drugs associated with *B. fragilis* were found by previously published literature. For example, 85% out of the top 20 predicted candidate drugs for *B. fragilis* are validated (Table 4); more details can be found in the Supplementary Material. These results of prediction demonstrate the ability of NNAN for predicting potential DMAs in practice.

**TABLE 4:** Top 20 predicted drugs associated with *Bacteroides fragilis.*

Drug name	Evidence	Drug name	Evidence
*NATEGLINIDE*	PMID: 17253883	*RAMIPRIL*	PMID: 31158845
*BENAZEPRIL*	PMID: 20445573	*DILTIAZEM*	unconfirmed
*VORICONAZOLE*	PMID: 18034666	*CLEMASTINE FUMARATE*	PMID: 31158845
*FEBUXOSTAT*	PMID: 18421623	*NAPROXEN (+)*	PMID: 15058617
*LOPERAMIDE*	PMID: 18192961	*ERGONOVINE MALEATE*	PMID: 17948937
*DIGITOXIN*	PMID: 1944247	*DROSPIRENONE*	PMID: 28986954
*SOTALOL*	PMID: 27836712	*DICYCLOMINE*	unconfirmed
*EZETIMIBE*	PMID: 15871634	*PROCARBAZINE*	PMID: 1316811
*IRBESARTAN*	PMID: 12800253	*RIZATRIPTAN BENZOATE*	unconfirmed
*SUMATRIPTAN SUCCINATE*	PMID:19925626	*SULPIRIDE*	PMID: 31158845

*The first column records the top 10 drugs, while the third column records the top 10–20 drugs.*

## Conclusion

This work has introduced NNAN, a deep learning-based bipartite graph network model to predict potential associations between drugs and microbes. NNAN calculates drug similarities using the weights of feature substructures. It provides an embedding representation based on the near neighbor aggregation for drug–microbe pairs, to enhance the explanation of DMAs. In addition, the model provides a crucial feature selection attention matrix for achieving more accurate predictions. These three components of NNAN jointly reveal that a drug associated with a specific microbe can always find its top-*l* near neighbors among other drugs that associate with the same microbe. Moreover, they uncover that the higher the ranks of its top-*l* near neighbors are, the more possible it is to associate with the microbe. Under both a cross-validation setting and a realistic potential linkage discovery setting, the empirical comparison of the proposed framework with three state-of-the-art baselines demonstrates that NNAN has significant competitive performance in predicting DMA. In addition, the framework of our model can also be evaluated in more similar biological issues (e.g., miRNA–disease, drug–target, and compound–protein associations prediction). Furthermore, there is still room to improve the model. We can set new experimental scenarios, which identify the DMAs for new drugs or new microbes, and can also integrate more biological databases to enrich the information of DMAs to improve the predictive ability.

## Data Availability Statement

The original contributions presented in the study are included in the article/Supplementary Material, further inquiries can be directed to the corresponding author/s.

## Author Contributions

J-YS and HY designed and supervised the study. BZ engaged in study design, drafted the manuscript, performed experiments, and analyzed data. YX coded and implemented the model, performed experiments. PZ assisted with performing experiments. S-MY assisted with supervising the study. All authors contributed to the article and approved the submitted version.

## Conflict of Interest

The authors declare that the research was conducted in the absence of any commercial or financial relationships that could be construed as a potential conflict of interest.

## Publisher’s Note

All claims expressed in this article are solely those of the authors and do not necessarily represent those of their affiliated organizations, or those of the publisher, the editors and the reviewers. Any product that may be evaluated in this article, or claim that may be made by its manufacturer, is not guaranteed or endorsed by the publisher.
