# Corrigendum: NNAN: Nearest Neighbor Attention Network to Predict Drug–Microbe Associations

**DOI:** 10.3389/fmicb.2022.944952

**Published:** 2022-05-30

**Authors:** Bei Zhu, Yi Xu, Pengcheng Zhao, Siu-Ming Yiu, Hui Yu, Jian-Yu Shi

**Affiliations:** ^1^School of Life Sciences, Northwestern Polytechnical University, Xi'an, China; ^2^Department of Computer Science, The University of Hong Kong, Hong Kong, China; ^3^School of Computer Science, Northwestern Polytechnical University, Xi'an, China

**Keywords:** deep learning, bipartite graph network, link prediction, drug-microbe association, attention matrix

In the original article, there was an error in “affiliation 1” as published. Instead of “School of Life Sciences, Northwestern Polytechnic University, Xi'an, China,” it should be “School of Life Sciences, Northwestern Polytechnical University, Xi'an, China.”

In the original article, there was an error in “affiliation 3” as published. Instead of “School of Computer Science, Northwestern Polytechnic University, Xi'an, China,” it should be “School of Computer Science, Northwestern Polytechnical University, Xi'an, China.”

The authors apologize for this error and state that this does not change the scientific conclusions of the article in any way. The original article has been updated.

## Publisher's Note

All claims expressed in this article are solely those of the authors and do not necessarily represent those of their affiliated organizations, or those of the publisher, the editors and the reviewers. Any product that may be evaluated in this article, or claim that may be made by its manufacturer, is not guaranteed or endorsed by the publisher.

